# Anisotropic Ferromagnetism in CrAu_3_Sb_6_

**DOI:** 10.1021/acs.chemmater.6c00999

**Published:** 2026-05-29

**Authors:** Michael A. McGuire, Nabaraj Pokhrel, Matthew S. Cook, Jiaqiang Yan, Andrew F. May, David Mandrus, David S. Parker

**Affiliations:** † Materials Science and Technology Division, 6146 Oak Ridge National Laboratory, Oak Ridge, Tennessee 37831, United States; ‡ Department of Materials Science and Engineering, 189503 The University of Tennessee, Knoxville, Tennessee 37996, United States

## Abstract

The crystal structure and properties of CrAu_3_Sb_6_ are presented, determined by measurements on single crystal and polycrystalline samples and first-principles calculations. The trigonal structure (space group *P*3̅1*m*) comprises a CdI_2_-like sublattice of AuSb_2_ with Cr occupying octahedral holes in a fully ordered triangular array. It can be viewed as a variation of the interesting and well-known families of partially intercalated transition metal dichalcogenides, but with stronger interactions along the stacking direction evidenced by short Cr–Au distances. The compound is metallic and ferromagnetic with a Curie temperature of 164 K. A strong anomalous contribution to the Hall effect is seen in the ferromagnetic state, and quantum oscillations are observed in magnetization at 2 K. Magnetization measurements show that the ordered moments of 1.5 μ_B_ per Cr are oriented along the *c*-axis with relatively strong magnetocrystalline anisotropy. Electronic structure calculations confirm this uniaxial anisotropy and the important role of spin–orbit coupling in CrAu_3_Sb_6_ and reveal strongly favored ferromagnetic ground state consistent with the measured Curie temperature. Through combined experiment and theory, this work provides a detailed picture of the basic properties and behaviors of this uniquely structured, Cr-based, anisotropic ferromagnet.

## Graphical abstract

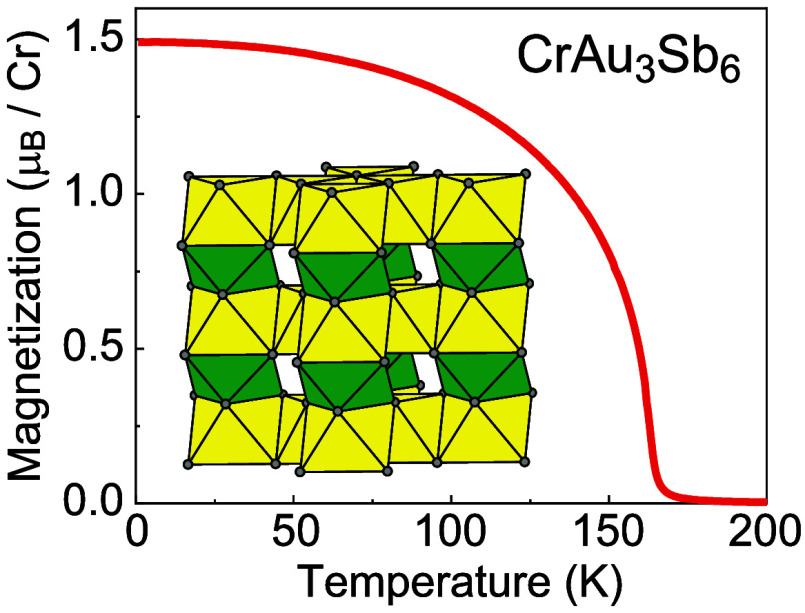

## Introduction

The main intrinsic source of magnetic anisotropy is spin–orbit coupling (SOC), which links electronic states to crystallographic symmetry and local coordination. This is referred to as magnetocrystalline anisotropy. SOC is a relativistic effect that increases strongly with atomic number *Z*, thus strong SOC is found in heavy elements. In magnetic materials, this provides the anisotropy required to realize certain quantum spin liquids,^1,2^ nontrivial spin textures,^3,4^ and strong permanent magnets.^5,6^ More generally, SOC links the chemical and physical properties in many important classes of materials and applications, including spintronics,^7–9^ thermoelectrics,^10,11^ and catalysis.^12,13^ In semiconductors, SOC is responsible for the band inversion that generates topological insulators.^14,15^ In superconductors, SOC affects pairing symmetry and can produce topological superconductivity.^16–18^

This presents a generally interesting and promising strategy for materials discovery and development: using crystal chemistry to combine strong SOC on one atom type with other targeted properties from other atoms or sublattices. The uniaxial ferromagnet CrI_3_ is a good example of this idea.^19^ While the moment on the heavy I^–^ is small, its strong SOC produces anisotropic superexchange interactions between magnetic Cr^3+^ atoms^20^ providing the anisotropy needed to maintain robust ferromagnetism in the 2D limit.^21^ Such ideas motivated the present investigation of the ternary Cr–Au–Sb system, in order to combine Cr’s magnetism with Au’s strong SOC. While binary compounds are known to form between each pair of elements (CrSb, CrSb_2_, AuSb_2_, Au_4_Cr), no ternary Cr–Au–Sb phases had been reported. This resulted in the discovery of the compound CrAu_3_Sb_6_. Its crystal growth, crystal structure, and properties are reported here.

As its composition may suggest, CrAu_3_Sb_6_ or Cr_1_/_3_AuSb_2_ is structurally related to the diverse and interesting family of partially intercalated transition metal dichalcogenides M_*x*_TQ_2_,^22^ where M is typically a 3d metal (V, Cr, Mn, Fe, Co, Ni, Cu), T is a transition metal often but not always from groups 4–6, and Q is a chalcogen. Several interesting behaviors have been seen in such compounds with $x=\frac{1}{3}$ , including helimagnetism and solitons,^23,24^ skyrmions,^25^ and nematic order.^26^ Spin-glass behavior has been reported in related phases with Cr and Fe intercalants.^27,28^

Measurements reported here of magnetization, electrical transport, and heat capacity show that CrAu_3_Sb_6_ is a metallic ferromagnet with relatively strong uniaxial magnetocrystalline anisotropy. It has a Curie temperature of 164 K and an anisotropy field near 67 kOe at 2 K. Density functional theory calculations are in reasonable agreement with measured behaviors, and these results highlight the importance of SOC on the electronic structure and magnetic properties.

## Results and Discussion

### Crystal Growth and Structure

CrAu_3_Sb_6_ was identified in the product of a reaction at nominal composition CrAuSb_2_. Powder X-ray diffraction of the multiphase product indicated the presence of an unknown compound, and magnetization measurements showed a ferromagnetic transition near 160 K that was determined to be associated with the unknown phase. Microstructural analysis using energy dispersive spectroscopy (EDS) in a scanning electron microscope showed that upon cooling a “CrAuSb_2_” composition, CrSb crystallized first and the unknown phase (EDS composition of Au_30_Cr_10_Sb_60_) formed around the CrSb grains. Finally, a fine two-phase mixture consistent with the eutectic on the Au–Sb binary phase diagram formed in the intergranular regions. Nearly single phase, polycrystalline samples were made by quenching a CrAu_3_Sb_6_ melt from 1050 °C and annealing at resulting boule at 430 °C for several days. EDS showed the sample to contain relatively large grains of CrAu_3_Sb_6_ with small pockets of AuSb_2_ and Au.

Differential scanning calorimetry measurements on a nearly single-phase sample showed a strong melting event centered around 460 °C. A sample heated through this event to 520 °C and cooled quickly to room temperature was seen to have fully melted and frozen into a spherical shape. X-ray diffraction showed that the phase had only partially reformed upon rapid cooling, with AuSb_2_, CrSb_2_, and CrSb making up the majority of the sample. This indicates incongruent melting of CrAu_3_Sb_6_, which is supported by the microstructure of the multiphase sample described above.

Using this information determined from the polycrystalline samples, a growth was designed to obtain single crystals of CrAu_3_Sb_6_. The microstructure suggested that Au–Sb eutectic should provide an appropriate flux for crystal growth. The published Au–Sb binary phase diagram^29^ gives the eutectic temperature of 357 °C near 37% Sb.

For the growth, a composition of Au/Cr/Sb = 12:1:12 (molar ratios) was used, that is, CrAu_3_Sb_6_ with a flux of composition Au/Sb = 9:6 (40% Sb). Elemental starting materials were loaded into Canfield crucible sets^30^ and sealed into silica glass ampules. The ampules were heated to 1050 °C over 10 h and held at that temperature for 24 h to obtain a homogeneous melt, then cooled to 460 °C. Crystallization was achieved by slowly cooling at 0.5 °C/h from 460 to 375 °C. At this temperature the remaining molten flux was decanted using a centrifuge. This produced blocky, metallic-black crystals of CrAu_3_Sb_6_ and AuSb_2_, distinguishable by their morphologies. All crystals used for characterization described below were confirmed to be the correct phase by EDS as well as diffraction measurements from facets to confirm orientations. Single crystal X-ray diffraction data were collected from a fragment from a flux-grown crystal. The structure was solved and refined giving excellent agreement factors (details in Tables 1 and 2) and is seen to describe well the powder diffraction results from the polycrystalline sample [Figure 1(d)]. The crystal structure is described in detail below.

**Figure 1:**
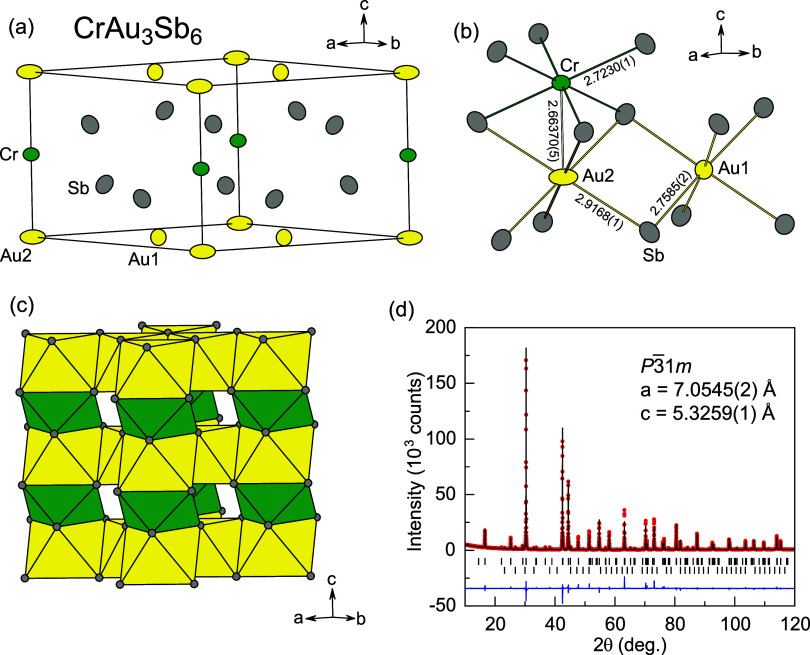
Crystal structure of CrAu_3_Sb_6_. (a) The unit cell with atoms shown using 95% probability. (b) Coordination environments for Cr and Au atoms. (c) Polyhedral view of the structure highlighting edge-sharing, Au-centered octahedra (yellow) joined by face sharing with strongly trigonally distorted Cr-centered octahedra (green). Panels (a–c) are based on the structure refined from single crystal X-ray diffraction data collected at room temperature. (d) The measured powder X-ray diffraction pattern from polycrystalline CrAu_3_Sb_6_ and Rietveld refinement using the structure determined from the single crystal data. The lower set of tick marks are associated with a small amount (∼5 wt %) of AuSb_2_ as an impurity.

**Table 1:** Crystal Structure Refinement Results for CrAu_3_Sb_6_ from Single Crystal X-ray Diffraction Collected at Room Temperature^a^

*a* (Å)	7.0547(1)
*c* (Å)	5.3274(1)
*V* (Å^3^)	229.616(8)
Data/parameters	763/14
*R*1 (all data)	0.0238
*wR*2 (all data)	0.0275
*x*_Sb_	0.3514(1)
*z*_Sb_	0.7115(1)

Nearest Interatomic Distances (Å)	
Au1–Sb	2.7585(1) [×6]
Au2–Sb	2.9168(2) [×6]
Cr–Sb	2.7230(2) [×6]
Cr–Au	2.66370(5) [×2]
Sb–Sb	3.0780(4) [×1]

a The space group is *P*3̅1*m*. Au1 is at Wyckoff position 2c ( $\frac{2}{3}$ , $\frac{1}{3}$ , 0), Au2 is at Wyckoff position 1a (0, 0, 0), Cr is at Wyckoff position 1b (0, 0, $\frac{1}{2}$ ), and Sb is at Wyckoff position 6k (*x*_Sb_, 0, *z*_Sb_).

**Table 2:** Anisotropic Displacement Parameters in Units of 10^–3^ Å^2^ for CrAu_3_Sb_6_ ^a^

	*U*_11_	*U*_22_	*U*_33_	*U*_23_	*U*_13_	*U*_12_	*U*_eq_
Au1	7(1)	7(1)	8(1)	0	0	4(1)	8(1)
Au2	21(1)	21(1)	6(1)	0	0	10(1)	16(1)
Cr	8(1)	8(1)	5(1)	0	0	4(1)	7(1)
Sb	9(1)	9(1)	10(1)	0	–2(1)	4(1)	9(1)

a The anisotropic displacement factor exponent takes the form: −2π^2^[*h* ^2^ a*^2^ *U*_11_ +... + 2hka*b*U_12_].

With the structure established, the expected thermodynamic phase stability was assessed by constructing a convex hull from total energies obtained via machine-learned interatomic potential (MLIP) relaxations, using the UMA-S-1.1 model^31^ and open-source Python code^32^ as described in the Methods section. CrAu_3_Sb_6_ is predicted to lie 20 meV/atom above the hull, with the nearest decomposition reaction being CrAu_3_Sb_6_ → Cr + 3AuSb_2_; no competing Cr–Au–Sb ternary appears in the computed phase diagram. The 20 meV/atom figure should be read in the context of the full Cr–Au–Sb hull, in which nearly every other compound, though experimentally established, is also predicted by UMA-S-1.1 to be metastable: CrAu_4_ at 17 meV/atom, AuSb_3_ at 23 meV/atom, CrSb_2_ at 83 meV/atom, and CrSb at 86 meV/atom. That is, the known binary chromium antimonides, both long-studied phases, are placed four times further above the hull by the same calculation than the compound under discussion. This pattern is consistent with the reported UMA-S test MAE of 20 meV/atom on the WBM benchmark^31^ and with the well-documented difficulty DFT-based methods have with the magnetic reference states of Cr-containing intermetallics, which can shift computed formation energies by 10–30 meV/atom depending on which magnetic ordering is assumed.^33–35^ Taken together, the MLIP-computed hull distance of 20 meV/atom places CrAu_3_Sb_6_ firmly in the synthesizability window established empirically by the other members of its own chemical system, and the successful synthesis is fully consistent with these calculations.

The crystal structure is shown in Figure 1(a–c). The gold atoms Au1 and Au2 together form a triangular net in the *ab*-plane. They are coordinated by Sb atoms in edge-sharing octahedra forming layers of composition AuSb_2_. The Au centered octahedra have slight trigonal distortions; the Sb–Au–Sb angles are 84.8, 91.4, 92.0, and 175.4° around Au1, and are 85.2, 94.8, and 180.0° around Au2. These layers are connected to one another by Cr filling 1/3 of the strongly distorted octahedral holes coordinated by three Sb atoms in each adjacent layer. Angles around Cr are 75.9, 104.1, and 180.0°. Bond distances are labeled on Figure 1(b).

The AA stacking sequence of the AuSb_2_ layers in CrAu_3_Sb_6_ produces a CdI_2_-like structure. Thus, the overall structure can be viewed as a partially Cr “intercalated” CdI_2_-type AuSb_2_ (Cr_1/3_AuSb_2_). This is reminiscent of similar phases that are common in transition metal dichalcogenides (TMD), although they generally have trigonal prismatic instead of octahedral coordination in the framework layers (MoS_2_-like instead of CdI_2_-like).^36^ It is not clear that direct structural analogue among TMDs is known, combining octahedral coordination of the metal ions in the close-packed layer with this ordered filling of 1/3 of the interstitial sites. The closest analogue may be Fe_1/3_VSe_2_, reported to have a CdI_2_-like framework of VSe_2_, but with variation on the in-plane intercalant order and stacking sequence.^37^

The observed nearest-neighbor Cr–Sb distance in CrAu_3_Sb_6_ is close to that seen in CrSb_2_ (2.69–2.74 Å)^38^ and slightly shorter than in CrSb (2.78 Å).^39^ The Au–Sb distances in this compound vary substantially between the two Au sites. The more isolated Au1 atoms have Au–Sb distances (2.76 Å) consistent with those seen in AuSb_2_ (2.76 Å),^40^ while the Au2 atoms adjacent to the Cr-centered octahedra have significantly longer bonds to Sb (2.92 Å). This discrepancy is likely related to Cr–Au2 interactions along the chains, with Cr–Au distances of 2.66 Å (=*c*/2). This is shorter than the distance of 2.89 Å in the only reported binary phase Au_4_Cr.^41^ Thus, it may be expected that some amount of Au–Cr bonding is responsible for the longer than expected Au–Sb distances around Au2 and the strong trigonal compression of the Sb octahedra around Cr. As a result of this interaction, an alternative description of the structure is an array of 1D chains of alternating Au2 and Cr in 6-fold coordination, with the chains running along the *c* direction and connected to neighboring chains through Au1 atoms. This interpretation is also apparent in the views of the structure shown in Figure 1.

The relatively large and anisotropic atomic displacement parameters (ADPs) for Au2 could be interpreted as evidence for either partial occupation of this site or splitting onto multiple nearby sites. However, no indication of partial occupancy on the Au2 site was seen. The Au occupancy of this site remained at 1.00 when refined as a mixture of Au and vacancies or as a mixture of Au and Cr. In addition, attempts to move Au2 off of its high symmetry site resulted in the atom returning to the origin or an unstable refinement. Thus, based on the present data, it appears that the best interpretation of the large ADPs for Au2 is that it indeed experiences larger and more anistotropic vibrations than other atoms in the structure. The observed anisotropy is surely related to the relatively short distance to Cr along *c* and relatively long distances to the coordinating Sb atoms. However, other possible explanations for the large ADPs cannot be ruled out at this time. These include static or dynamic disorder over slightly offset positions that cannot be resolved by our X-ray data including the possibility of short-range order. More could likely be learned from temperature dependent ADP measurements.

Assigning typical oxidation states of +1 for Au and +3 for Cr gives a total cationic charge per formula unit of +6. Enforcing charge balance then gives Sb^1–^. This is inconsistent with the anionic framework in CrAu_3_Sb_6_, which contains at most weakly bound dimers of Sb (*d*_Sb–Sb_^min^ = 3.08 Å). In the Zintl picture, dimerized Sb would have a charge of −2 per Sb; realizing a formal charge of −1 would require a two-bonded Sb structure, like the square rings in skutterudite CoSb_3_. Alternatively, treating the Sb as dimerized gives a formal anionic charge of −12 per formula unit. This could be balanced by a +3 state on both Cr and Au, but stabilizing Au^3+^ is not reasonable in an antimonide. These inconsistencies suggest that CrAu_3_Sb_6_ should be treated as an intermetallic compound and should display metallic transport properties, which are supported by the physical properties and electronic structure calculations discussed in the following sections.

### Physical Properties

Temperature and field dependent magnetization, heat capacity, and electrical transport properties were measured on flux-grown single crystals as well as polycrystalline samples. Figure 2 summarizes the behavior of polycrystalline CrAu_3_Sb_6_. A clear indication of a phase transition is seen near 160 K. At this temperature, the resistivity shows a sharp drop upon cooling [Figure 2(a)], the heat capacity shows a lambda-like anomaly [Figure 2(b)], and the magnetization shows a strong increase upon cooling [Figure 2(c)]. This suggests a transition to a ferromagnetic state at low temperature. This is confirmed by isothermal magnetization measurements at 2 K [Figure 2(d)]. A gradual increase toward a saturation moment is observed along with magnetic hysteresis. These observations are consistent with a polycrystalline ferromagnet with significant intrinsic (magnetocrystalline) anisotropy and some amount of domain wall pinning producing coercivity.

**Figure 2:**
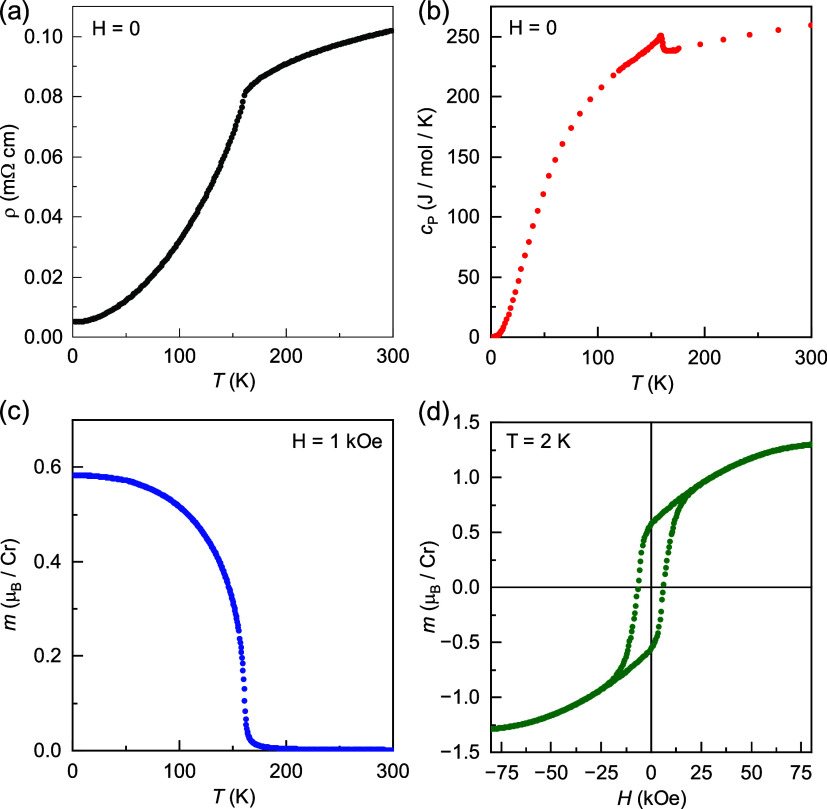
Temperature and field dependent physical properties of polycrystalline CrAu_3_Sb_6_, including (a) the resistivity ρ, (b) heat capacity *c*_P_, and (c, d) magnetization *m*. The data were measured using samples cut from dense cast and annealed material that was nearly single phase.

A more detailed analysis and discussion of the low temperature behavior of CrAu_3_Sb_6_ based upon data from flux-grown single crystals follows, since single crystal data give better insight into intrinsic behaviors and anisotropies. The magnetization, heat capacity, and electrical transport data from the crystals are consistent with those of the polycrystalline samples.

#### Magnetization

The temperature dependence of the magnetization measured in an applied field of 1 kOe both parallel and perpendicular to the crystallographic *c*-axis are shown in Figure 3(a). The anisotropy suggested by the polycrystalline data is seen, with a much stronger and clearly ferromagnetic response observed with the field along *c*. With the field in the plane, the response is relatively weak and shows a cusp where the magnetization along *c* rises sharply. This demonstrates that CrAu_3_Sb_6_ is a uniaxial ferromagnet with its easy axis along *c*. Data collected at a lower field (100 Oe) along the *c*-axis and near the transition temperature is shown in Figure 3(c). From this, a Curie temperature of 163.5 K is determined from the minimum in the temperature derivative of the magnetization.

**Figure 3:**
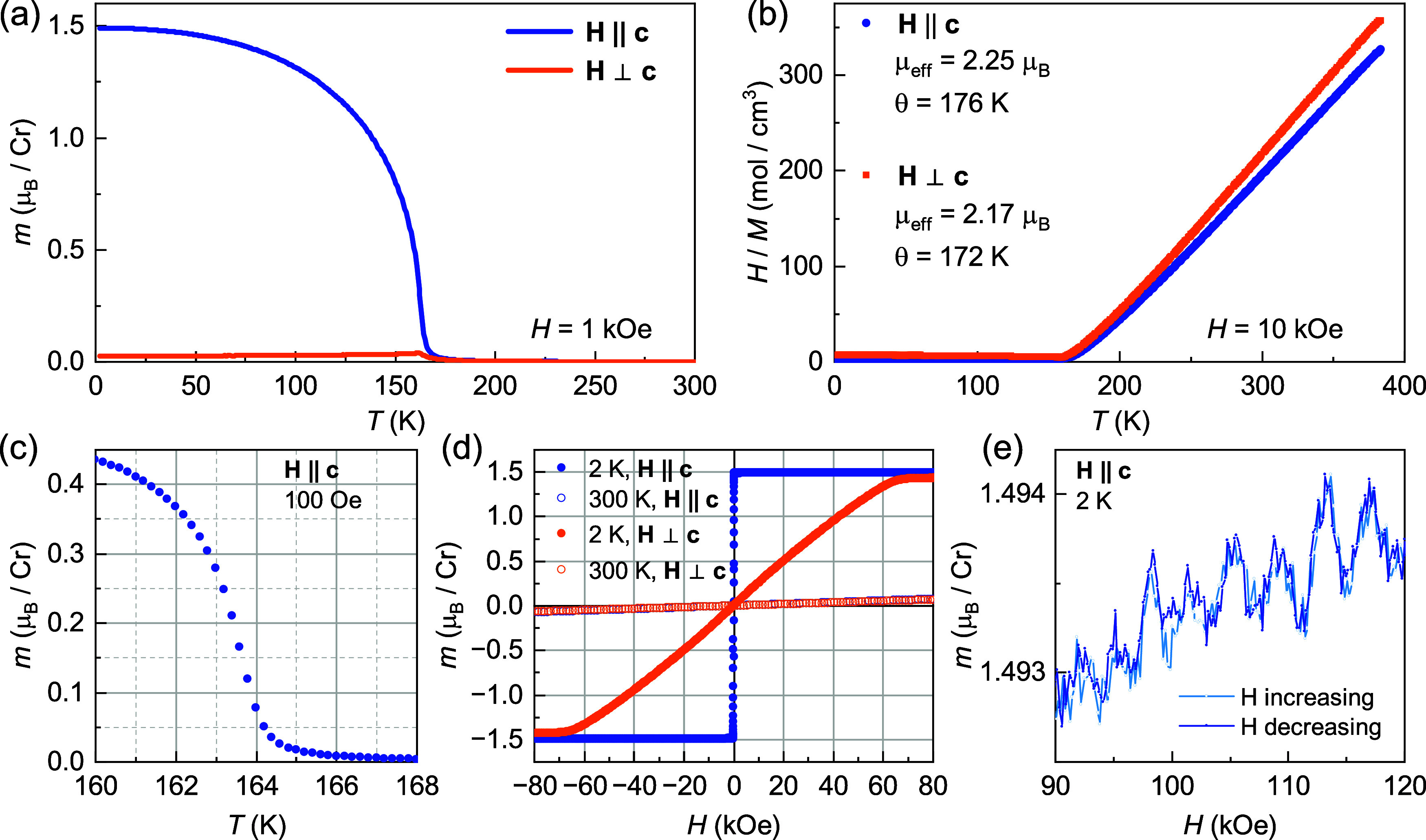
Magnetization data collected on single crystal CrAu_3_Sb_6_. Data are shown for magnetic fields applied both along the *c* axis (∥*c*) and in the ab-plane (⊥*c*). (a) Temperature dependence of the magnetization measured at 1 kOe. (b) Temperature dependence of the inverse magnetic susceptibility (*H*/*M*) measured at 10 kOe, with results from Curie–Weiss fits for data above 250 K listed on the plot. (c) Low-field magnetization data near the Curie temperature of 164 K. (d) Isothermal magnetization loops measured at 2 and 300 K. (e) Field dependence at high field and low temperature measured on both increasing and decreasing field showing evidence of quantum oscillations.

Above *T*_C_, the magnetization shows Curie–Weiss behavior expected for a local-moment paramagnet. Inverse susceptibility versus temperature is shown in Figure 3(b). Results of linear fits to data between 250 and 375 K are included on the plot. The results show nearly isotropic behavior in the paramagnetic state, with Weiss temperatures (θ) that are positive and close to the Curie temperature and effective moments (μ_eff_) of about 2.2 μ_B_. This is significantly smaller than the value of 3.87 μ_B_ seen in chromium’s typical trivalent oxidation state (3d^3^, *S* = 3/2), and in fact falls between the spin-only values expected for *S* = 1/2 and 1 (1.73 and 2.83 μ_B_, respectively). This may be expected due to the intermetallic nature of this compound.

The measured saturation magnetization at 2 K [Figure 3(d)] is 1.5 μ_B_/Cr. This is lower than expected for trivalent Cr, consistent with the paramagnetic effective moment at high temperature. These can be compared by calculating the effective spin value that corresponds to each moment. For the saturation moment *M*_S_ and paramagnetic effective moment μ_eff_, the effective spin *S*_eff_ is given by the relations *M*_S_ = *gS*_eff_ and $\mu_{\mathrm{eff}}=g\sqrt{S_{\mathrm{eff}}\left(S_{\mathrm{eff}}+1\right)}$ . For a “normal” local moment on an ion, *S*_eff_ determined by these two relationships is the same and is equal to half the total number of unpaired spins. Data for CrAu_3_Sb_6_ give *S*_eff_ values of 0.71 from the Curie–Weiss fit and 0.75 from the saturation magnetization. The ratio is 0.95. This shows that the measured saturation moment is consistent with the moment determined from fitting in the paramagnetic regime, indicating behavior generally consistent with local moment magnetism. This ratio is known as the Rhodes–Wohlfarth ratio and is used to distinguish local moment ferromagnets, where the ratio is close to one, from itinerant magnets, where the ratio is typically much larger than one.^42^

The magnetic anisotropy is demonstrated by isothermal magnetization loops collected at 300 and 2 K [Figure 3(d)]. The most notable feature is the relatively large anisotropy field at low temperature. Magnetization along the *c* axis saturates abruptly at fields below 500 Oe. Magnetization perpendicular to the *c* axis saturates near 67 kOe. The magnetocrystalline anisotropy constant can be calculated from this using $K=\frac{1}{2}H_{A}M_{S}$ , where *H*_A_ = 67 kOe is the anisotropy field and *M*_S_ = 60.4 emu/cm^3^ is the saturation magnetization. This gives *K* = 2.0 × 10^6^ erg/cm^3^ or 0.29 meV/Cr. These parameters describing the magnetic behavior of CrAu_3_Sb_6_ are discussed below in the context of first-principles electronic structure and energy calculations.

At high fields and low temperature, relatively weak but distinct De Haas–Van Alphen oscillations were observed. This is shown in Figure 3(e). The data are somewhat noisy, but measurements on increasing and decreasing field show consistent oscillations confirming their intrinsic nature. Due to their small amplitude and the relatively small range of field over which they are observed in these crystals, further analysis of the oscillations and connections to the electronic structure are left for future studies.

The observed magnetic behavior of CrAu_3_Sb_6_ can be compared to other chromium compounds. Interestingly, several quasi-2D ferromagnets of interest to the van der Waals heterostructure field are based on chromium. This includes the trihalides CrBr_3_ (*T*_C_ = 37 K^43^) and CrI_3_ (*T*_C_ = 61 K^19^), the ternary tellurides CrSiTe_3_ (*T*_C_ = 33 K^44^) and CrGeTe_3_ (*T*_C_ = 61 K^45^). All of these compounds are uniaxial ferromagnets. Unlike CrAu_3_Sb_6_, they all have ordered moments near 3 μ_B_ per Cr, which is consistent with their formally trivalent Cr ions and their semiconducting or insulating natures. The strongest magnetic anisotropy is found in CrI_3_, with an anisotropy field of 30 kOe at 2 K. The data in ref 19. (*M*_S_ = 210 emu/cm^3^, *H*_A_ = 30 kOe) gives an estimate of *K* = 3.2 × 10^6^ erg/cm^3^ or 0.27 meV/Cr, similar on a per-Cr basis to K estimated for CrAu_3_Sb_6_ here. All of these compounds have Curie temperatures significantly lower than CrAu_3_Sb_6_, which may be related to their lower dimensionality.

Somewhat similar structural analogues for CrAu_3_Sb_6_ are found among the TMDs with ordered Cr intercalants, typified by Cr_1/3_NbS_2_ (CrNb_3_S_6_). This compound includes close packed trilayers of S–Nb–S analogous to the AuSb_2_ layers in CrAu_3_Sb_6_; however, these trilayers have an ABA stacking, giving trigonal prismatic coordination to Nb, unlike the octahedral coordination of Au. The gaps between neighboring NbS_2_ trilayers have 1/3 of their octahedral holes filled with Cr atoms in a triangular net like in CrAu_3_Sb_6_. The magnetic sublattices in these compounds are then formed by stacking these triangular Cr nets. In Cr_1/3_NbS_2_ ^23^ and Cr_1/3_TaS_2_ ^24^ the Cr layers are staggered in an AB stacking arrangement. These compounds have helimagnetic ground states. In Cr_1/3_NbSe_2_ ^46^ the Cr layers adopt an AA stacking like that seen in CrAu_3_Sb_6_. This compound has a ferromagnetic ground state. Gubkin et al. in ref 46. note this relationship between the stacking sequence and associated inversion symmetry breaking and the magnetic ground states in these materials. A noteworthy distinction between these compounds and CrAu_3_Sb_6_ is that they all have planar anisotropy. Still, it is interesting to note that the AA stacking of Cr in CrAu_3_Sb_6_ also supports simple ferromagnetic order.

#### Heat Capacity

The results of heat capacity measurements are summarized in Figure 4, with data over the full temperature range measured, 1.8–250 K, shown in Figure 4(a). At 250 K, *c*_P_ reaches 252 J·K^–1^ mol^–1^, close to the expected Dulong–Petit value of 249 J·K^–1^ mol^–1^. The anomaly at the Curie temperature is clearly seen. This is shown on a smaller scale in Figure 4(b), where the onset of a sharp λ-like anomaly is observed at 163.5 K. Data collected at an applied magnetic field of 10 kOe is shown for comparison. The field strongly broadens the heat capacity feature, as expected for a ferromagnetic transition. An estimate of the entropy associated with the anomaly was determined by subtracting a smooth background fit to data above 167 K and below 137 K, and integrating the resulting *c*_P_/*T* vs *T* curve (not shown). This gave a value near 1.2 J·K^–1^ mol^–1^, only about 20% of *R* ln 2. This suggests entropy is released over a wider temperature range, and a better estimate of the lattice and electronic heat capacity would be needed to probe this more rigorously.

**Figure 4:**
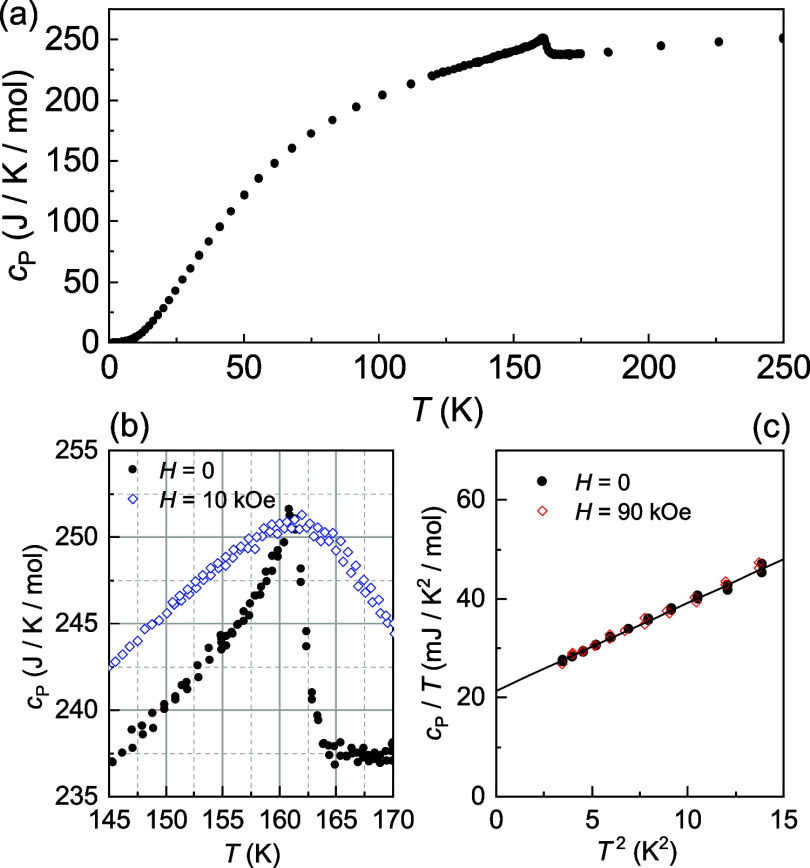
Results of heat capacity measurements on CrAu_3_Sb_6_ crystals. (a) The measured heat capacity between 1.8 and 250 K. (b) Heat capacity near the phase transition showing the sharp lambda anomaly in zero field and the broadening of the anomaly in moderate applied fields (10 kOe shown). (c) Low temperature behavior and linear fits used to extract the Sommerfeld coefficient and Debye temperature estimates. The unit mol here and throughout the paper refers to mole of formula units or equivalently mole of Cr atoms unless explicitly stated otherwise.

Low temperature heat capacity is shown in Figure 4(c). This includes data collected at zero field and at 90 kOe, plotted as *c*_P_/*T* vs *T* ^2^. The magnetic field has no measurable effect over this temperature range. The linear fit shown on the plot gives a Sommerfeld coefficient of γ = 21 mJ mol^–1^ K^–2^, supporting significant DOS at the Fermi level and metallic behavior, and a Debye temperature of Θ_D_ = 220 K. This assumes that any contribution from magnetic excitations to the heat capacity are negligible over this temperature range (*T* < 4 *K* ≈ 0.025*T*_C_), which is likely true. The Debye temperature can be estimated from the higher temperature data [Figure 4(a)], using the fact that *c*_Debye_(*T* = Θ_D_/2) ≈ 3*R*/2. This gives a consistent estimate of Θ_D_ = 200 K.

#### Electrical Transport

Electrical transport data for CrAu_3_Sb_6_ are collected in Figure 5. Data are shown for current flowing in both the *c* direction and the *ab* plane. The anisotropy is generally small, but does reach a factor of about two at 2 K. Overall the resistivity (ρ) increases with temperature up to 300 K as expected for a metal, but the temperature dependence changes dramatically across the Curie temperature. Above *T*_C_ the temperature dependence is relatively weak and concave. This is abnormal behavior for a metal, and suggests something beyond electron phonon scattering dominates the transport at high temperatures. Sources may include scattering from magnetic fluctuations above *T*_C_, activation of additional carriers or contributions from additional bands as temperature rises, or approach to the Mott-Ioffe-Regel resistivity limit. This limit is approached as the mean free path decreases toward the limiting value of one lattice spacing, and is often in the 100–300 μΩ·cm range.^47,48^

**Figure 5:**
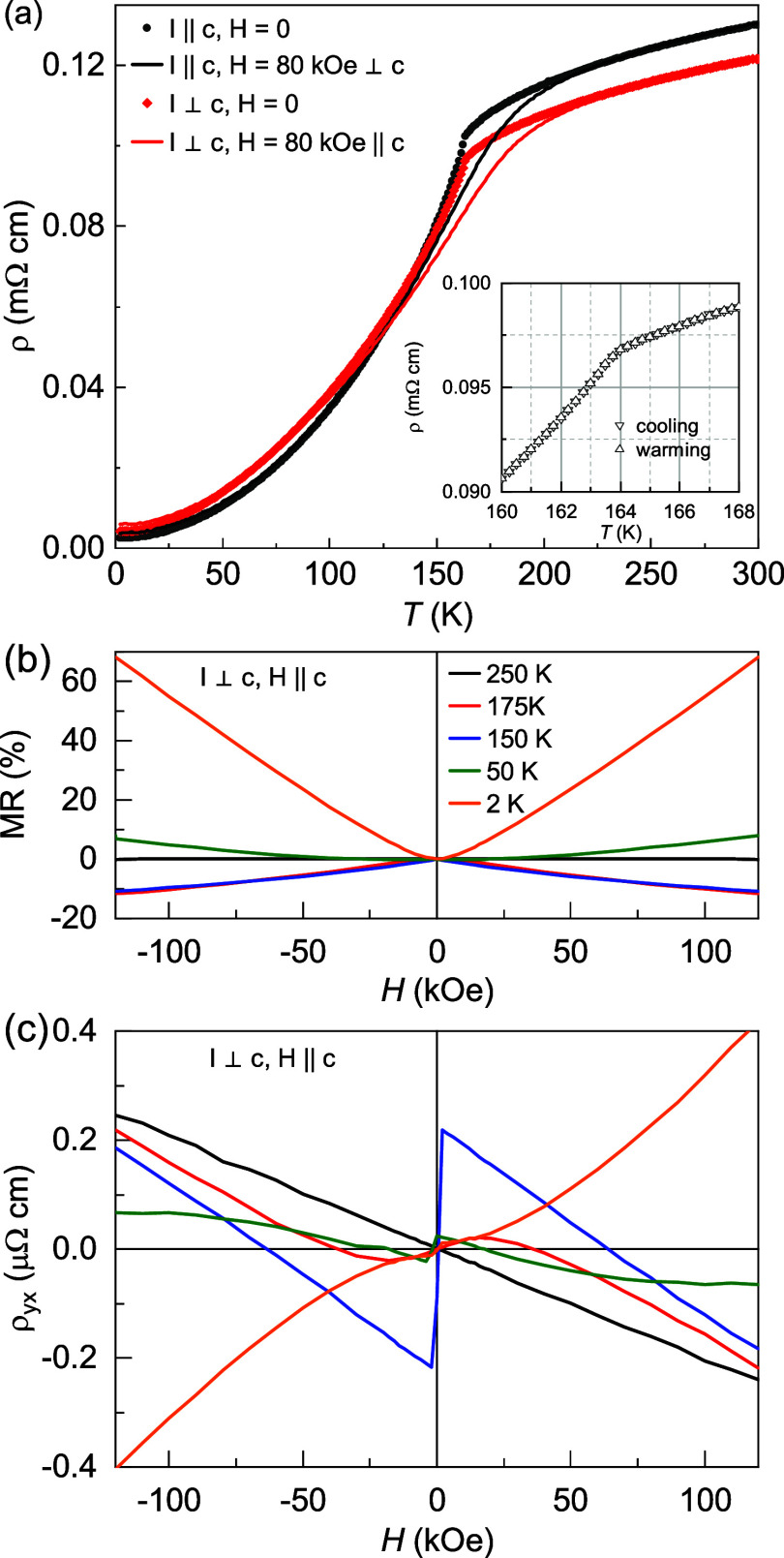
Electrical transport data for CrAu_3_Sb_6_ crystals. (a) Longitudinal resistivity measured with current along both the *c* direction and in the and *ab* plane (⊥*c*). Effects of fields applied perpendicular to the current flows are shown. The inset highlights the behavior near the phase transition (at zero field) and includes data collected on heating and warming revealing little or no thermal hysteresis. (b, c) Magnetoresistance, defined as *MR* = [*R*(*H*) – *R*(0) ]/*R*(0) and Hall resistivity measured well above, just below, and well below the Curie temperature.

Below *T*_C_ the temperature dependence of ρ is more similar to a normal metal, decreasing strongly on cooling to a plateau near 2–4 μΩ·cm. This gives a resistivity ratio ρ­(300 K)/ρ­(2 K) of 30 and 50 for with the current parallel and perpendicular to *c*, respectively. The inset of Figure 5(a) shows ρ measured along in the *ab*-plane upon both heating and cooling through *T*_C_. No thermal hysteresis is observed, consistent with that a second order phase transition. Upon cooling through *T*_C_, the resistivity downturn begins at 164 K, and the maximum in dρ/d*T* occurs at 163 K.

The temperature dependence measured in an applied field of 80 kOe is also shown in Figure 5(a). As expected, there is significant magnetoresistance near *T*_C_, with the suppression in ρ upon cooling pushed to higher temperatures when the field is applied. This is consistent with the ferromagnetic nature of the transition, and the heat capacity behavior noted above. Several isothermal transverse magnetoresistance (MR) curves with the field along the *c* axis are shown in Figure 5(b). The MR is very small at 250 K and becomes moderate and negative near *T*_C_, likely due to suppression of magnetic fluctuations. Upon further cooling MR changes sign and becomes strongly positive, reaching 70% in a field of 120 kOe at 2 K.

Figure 5(c) shows Hall effect results. At 250 K the data are linear and the Hall coefficient is negative. This may indicate transport dominated by electrons, but this can be an overly simplified interpretation in multiband metals. Like the MR, the ordinary part of the Hall effect changes sign at low temperature. An anomalous Hall effect appears below *T*_C_, as expected in this easy-axis ferromagnet. In the ρ_*yx*_ data this is most apparent at 150 K, where ρ_*xx*_ is high. The anomalous contribution to the Hall conductivity (σ_*xy*_ = ρ_*yx*_/(ρ_*yx*_^2^ + ρ_*xx*_^2^)) increases upon cooling, with values near −30, −140, and −600 S/cm at 150, 50, and 2 K, respectively. This increase upon cooling is tied to the increase in magnetization and longitudinal conductivity (σ_*xx*_ = 1/ρ_*xx*_) upon cooling. Modeling the temperature dependence in more detail may reveal information about the underlying mechanism but would require additional measurements and analysis.

### First-Principles Calculations

#### Electronic Structure

This material has a relatively small concentration of Cr yet supports ferromagnetism with significant anisotropy and a moderately high Curie temperature. We pursued first-principles calculations to probe the magnetism in CrAu_3_Sb_6_ and how spin orbit coupling from the heavy Sb and Au components might impact the magnetic properties.

Calculated densities of states (DOS) for both a nonmagnetic (NM) model and a ferromagnetic (FM) model with Cr spins aligned along the *c*-axis are shown in Figure 6. Spin orbit coupling (SOC) is included in both cases. As illustrated in Figure 6(a), the electronic states near and at the Fermi level in CrAu_3_Sb_6_ are predominantly derived from Sb and Cr. In the nonmagnetic calculation, a relatively large DOS is present at the Fermi level (*E*_F_), with the dominant contributions arising from Cr 3d and Sb 5p states. Such a high DOS near *E*_F_ indicates an electronic instability of the nonmagnetic phase and suggests a tendency toward magnetic ordering. Taking the transition metal DOS of *D*(*E*_F_) = 4 eV^–1^ Cr^–1^ from Figure 6a and the Stoner parameter of *I* = 0.77 for Cr,^6^ the Stoner criterion for ferromagnetism *D*(*E*_F_)*I* > 1 is clearly met.

**Figure 6:**
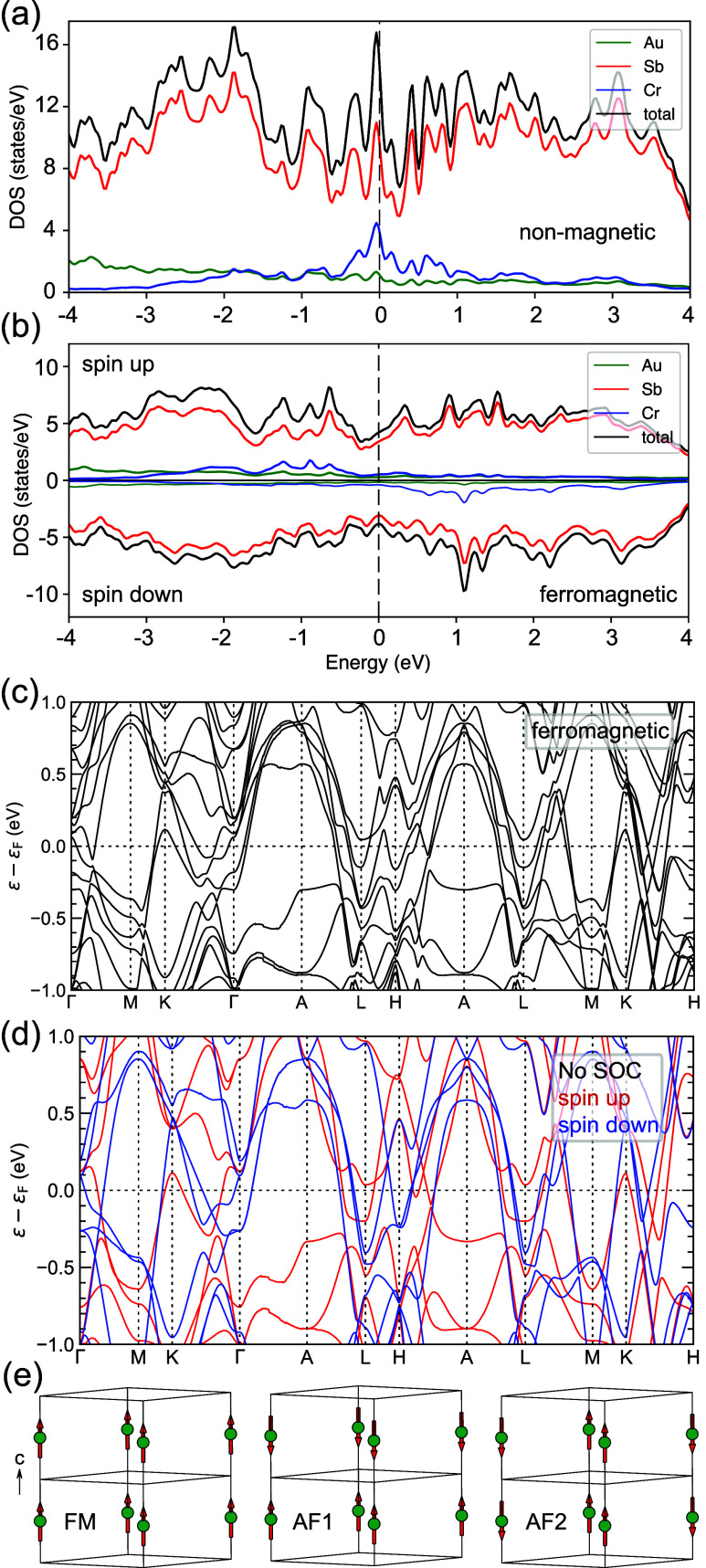
DFT results for CrAu_3_Sb_6_. The total and element-projected DOS for CrAu_3_Sb_6_ calculated (a) for nonmagnetic Cr ions and (b) for ferromagnetically aligned Cr ions with moments along the *c*-axis. Spin orbit coupling is included in both cases. (c) Bands near the Fermi level (ϵ_F_) corresponding to the DOS in (b). (d) Spin up and spin down bands from a calculation with no SOC. (e) Ferromagnetic (FM) and antiferromagnetic (AF) Cr moment configurations used in the calculations.

When ferromagnetism is included on the Cr sublattice, the DOS undergoes a pronounced exchange splitting between majority and minority spin channels, leading to a substantial suppression of the DOS at *E*_F_. This redistribution of electronic states lowers the total energy and stabilizes the ferromagnetic ground state, consistent with a Stoner-type mechanism of itinerant magnetism. Figure 6(b) presents the DOS for the ferromagnetic configuration with Cr moments aligned along [001], the experimentally determined easy axis.

The computed ordered magnetic moment of Cr is 1.93 μ_B_, while Au and Sb carry much smaller moments of 0.02 and 0.01 μ_B_, respectively. The calculated moment of Cr is about 30% larger than the experimentally measured saturation moment at 2 K, but is still reduced from local moment expected for Cr^3+^. Note that GGA + *U* calculations including SOC with *U* = 3 eV for the Cr d orbitals generated an even larger moment (3.14 μ_B_) and planar anisotropy, showing worse agreement with experiment.

While the moments on Au and Sb are small, their spin–orbit coupling is expected to contribute strongly to the anisotropy (see below), and the spin and element projected DOS curves in Figure 6(b) indicate these elements do participate in the magnetism and hybridize strongly with the magneic Cr atoms. In particular, where strong spin polarized peaks in the Cr DOS occur (e.g., about −0.7 and 1.0 eV), corresponding spin polarized peaks are seen in the other elements. Also, the Au DOS at the Fermi energy shows spin polarization, with 0.27 states/eV in the up channel and 0.15 states/eV in the down channel.

To better describe and understand the magnetism in this material, additional first-principles calculations were focused on addressing two rather unusual magnetic properties exhibited here. The first is the comparatively high observed Curie temperature of 164 K (considering the small atomic fraction, 1/10, of the magnetic atom Cr). The second is the relatively high uniaxial magnetic anisotropy field of ∼67 kOe observed in CrAu_3_Sb_6_ crystals, which results in a low-temperature coercivity as high as 6.5 kOe in the polycrystalline samples.

To address the Curie point, we have conducted total energy calculations of two magnetic states in addition to the ferromagnetic one: a state with the Cr planes alternating in orientation along the *c*-axis (AF1), and an additional state AF2 with 4 of the 6 planar Cr neighbors antialigned to the central Cr atom, the maximum possible in this hexagonal and thereby partially frustrated system (Figure 6e). In this latter calculation the nearest neighbor *c*-axis Cr–Cr orientation is parallel. Much previous work of ours, as well as basic mean-field interaction physics, has found that a good estimate of the magnetic ordering point can be made as Δ*E*/3, with Δ*E* being the energy difference, in K, between the ground state and an excited state based on the nearest-neighbor, or strongest exchange interaction.^49–54^ From direct calculation we find the AF1 nearest-neighbor state to lie some 37.8 meV/Cr above the ferromagnetic ground state, and the AF2 state 29.7 meV/Cr above the ferromagnetic state. The AF1 state energetics would suggest, using the Δ*E*/3 criterion, an ordering point of some 146 K, in good agreement with the experimental value of 164 K. Moreover, the density of states reveals pronounced hybridization between the Cr 3d and Sb 5p orbitals in the vicinity of the Fermi level. Such intralayer hybridization promotes electronic coupling among Cr 3d states, thereby strengthening the effective exchange interactions within the Cr sublattice. This enhanced exchange coupling likely underlies the sizable Curie temperature observed experimentally, in agreement with our first-principles calculations.

The first-principles calculations reproduce the observed uniaxial magnetic anisotropy along the [001] crystallographic direction seen experimentally. From them, a magnetic anisotropy energy of *K* = 0.21 meV/Cr atom is determined by calculating the energy cost of rotating the moments into a perpendicular direction. This is similar to but smaller than the experimentally determined estimate of 0.29 meV/Cr based on the isothermal magnetization curves at 2 K (see above). One can speculate that the strongly distorted octahedral environment contributes to this anisotropy, and to the difference between uniaxial CrAu_3_Sb_6_ and the easy-plane magnetism in the structurally related dichalcogenide based materials described above. This anisotropy is generated directly from spin–orbit coupling. Figure 6(c),(d) show how SOC affects the bands near the Fermi level by mixing spin-up and spin-down bands and opening gaps at band crossings, notably in the Γ–*M*–*K*–Γ and *A*–*H*–*L*–*A* planes.

A series of calculations were also performed with SOC included for each of the atom types individually. Interestingly, the sum of the separate MAE contributions calculated in this way (−0.45 meV/Cr) differs significantly in magnitude and sign from the calculation with SOC on all atoms simultaneously (0.21 meV/Cr). This highlights the important role of hybridization between atomic orbitals, where the anisotropy arises from the collective electronic structure including nonlocal and cooperative effects rather than isolated atomic terms.

## Summary and Conclusions

The crystal structure of CrAu_3_Sb_6_ is reminiscent of those adopted by Cr intercalated transition metal dichalcogenides, but with a CdI_2_-like (1T) substructure. Strong trigonal compression of the Cr-centered Sb octahedra in CrAu_3_Sb_6_ results in relatively short distances between Cr and Au atoms. This provides an alternative view of the structure as 1D Cr–Au chains, with chains connected to one another through Sb atoms bonded to additional, interchain Au sites. This distortion and chain-like structure likely determines the magnetic anisotropy, which is relatively strong and uniaxial. The ferromagnetic transition is observed in magnetization, transport, and heat capacity measurements. A Curie temperature of 164 K is determined from magnetization data. First-principles calculations confirm this uniaxial anisotropy arising from spin–orbit coupling and reveal strongly favored ferromagnetism supporting the relatively high *T*_C_. The band structure as well as electrical resistivity and heat capacity measurements show CrAu_3_Sb_6_ to be metallic, and a strong anomalous contribution to the Hall effect is seen in the ferromagnetic state. Relatively weak but clear quantum oscillations are observed in magnetization at 2 K. The calculated band structure is complicated, with many bands crossing the Fermi level. This supports the highly metallic behavior in resistivity (below *T*_C_) and precludes any simple interpretation of the oscillations. Overall, this work provides a detailed picture of the basic properties and behaviors of CrAu_3_Sb_6_, a unique and interesting Cr-based ferromagnet, and introduces a crystal chemistry with features that may lead to versatile magnetic behaviors combined with metallic transport, based on analogy with TMD-based materials.

## Methods

Synthesis methods are described in the main text. Compositional analysis by energy dispersive spectroscopy was performed using a Oxford Aztec spectrometer on a Hitachi TM-4000Plus tabletop scanning electron microscope. Powder X-ray diffraction employed a Malvern/PANalytical Empyrean powder diffractometer with monochromatic Cu Kα1 radiation. Rietveld analysis was performed using Fullprof.^55^ Single crystal X-ray diffraction employed a Bruker D8 Advance Quest diffractometer (Mo–Kα radiation), with crystals mounted on the edge of a kapton loop with a small amount of Parabar oil. Data were reduced and analyzed using tools in the Bruker Apex-4 software^56^ including semiempirical absorption corrections, and structures were refined using ShelX.^57^ Magnetization, heat capacity, and transport measurements were measured using standard practices with commercial cryostats from Quantum Design (Physical Property Measurement System and Dynacool).

First-principles calculations were performed within the framework of density functional theory (DFT)^58^ using the all-electron WIEN2k package.^59^ In WIEN2k, all electrons are treated explicitly in a fully self-consistent manner using the linearized augmented plane-wave (LAPW) method.^60–62^ The exchange–correlation interaction is described within the generalized gradient approximation (GGA) employing the Perdew–Burke–Ernzerhof (PBE) functional.^63^ Atomic positions were fully relaxed until the residual forces on all atoms were reduced to 1 mRy/Bohr or less. Muffin-tin sphere radii of 2.55, 2.44, and 2.28 Bohr were used for Au, Sb, and Cr, respectively, together with a plane-wave cutoff defined by RK_max_ = 9.0. Brillouin-zone integrations were carried out using 1000 *k*-points in the full Brillouin zone. For improved accuracy, the magnetic anisotropy energies were evaluated using a denser mesh of 5000 *k*-points.

For MLIP calculations, candidate structures for all competing phases in the relevant chemical system were first downloaded as CIF files from the Materials Project database. Each structure was then fully relaxed (atomic positions and cell parameters) using the Atomic Simulation Environment (ASE) with the FAIRChem UMA-small model (uma-s-1p1) serving as the energy/force calculator.^31,64^ Structural relaxations were performed with a force convergence criterion of 0.05 eV/Å. The relaxed total energies were used to construct a pymatgen PhaseDiagram object, from which the convex hull and energies above the hull were computed. The workflow is available as open-source Python code.^32^
